# Correlation Analysis of Different Measurement Places of Galvanic Skin Response in Test Groups Facing Pleasant and Unpleasant Stimuli

**DOI:** 10.3390/s21124210

**Published:** 2021-06-19

**Authors:** Andres Sanchez-Comas, Kåre Synnes, Diego Molina-Estren, Alexander Troncoso-Palacio, Zhoe Comas-González

**Affiliations:** 1 Department of Productivity and Innovation, Universidad de la Costa, 080 002 Barranquilla, Colombia; atroncos1@cuc.edu.co; 2 Department of Computer Science, Electrical and Space Engineering, Luleå Tekniska Universitet, 971 87 Luleå, Sweden; 3 Department of Computer Science and Electronics, Universidad de la Costa, 080 002 Barranquilla, Colombia; dmolina@cuc.edu.co (D.M.-E.); zcomas1@cuc.edu.co (Z.C.-G.)

**Keywords:** stress, wearable, sensor, physiological signals, galvanic skin response, GSR, electrodermal activity, EDA, pleasant and unpleasant stimuli, valence, correlation

## Abstract

The galvanic skin response (GSR; also widely known as electrodermal activity (EDA)) is a signal for stress-related studies. Given the sparsity of studies related to the GSR and the variety of devices, this study was conducted at the Human Health Activity Laboratory (H2AL) with 17 healthy subjects to determine the variability in the detection of changes in the galvanic skin response among a test group with heterogeneous respondents facing pleasant and unpleasant stimuli, correlating the GSR biosignals measured from different body sites. We experimented with the right and left wrist, left fingers, the inner side of the right foot using Shimmer3GSR and Empatica E4 sensors. The results indicated the most promising homogeneous places for measuring the GSR, namely, the left fingers and right foot. The results also suggested that due to a significantly strong correlation among the inner side of the right foot and the left fingers, as well as the moderate correlations with the right and left wrists, the foot may be a suitable place to homogenously measure a GSR signal in a test group. We also discuss some possible causes of weak and negative correlations from anomalies detected in the raw data possibly related to the sensors or the test group, which may be considered to develop robust emotion detection systems based on GRS biosignals.

## 1. Introduction

In recent decades, advances in data analytics techniques and sensor hardware development have led to the emergence of new research fields such as activity recognition, taking advantage of technologies such as smartphones, wearable devices, Internet of Things, and any device with sensors or embedded systems and digital storage or streaming capacity [1]. All of this has generated a wide range of new applications, from computer science to other fields such as elderly independent living [2,3], treatment of cognitive diseases [4], autism lifestyles [5], and care [6], at which this work is motivated. Human activity recognition (HAR) improves these fields, supported by specialized research and development on posture, gesture, localization, occupancy, and emotion recognition [7]. 

**Figure 1:**
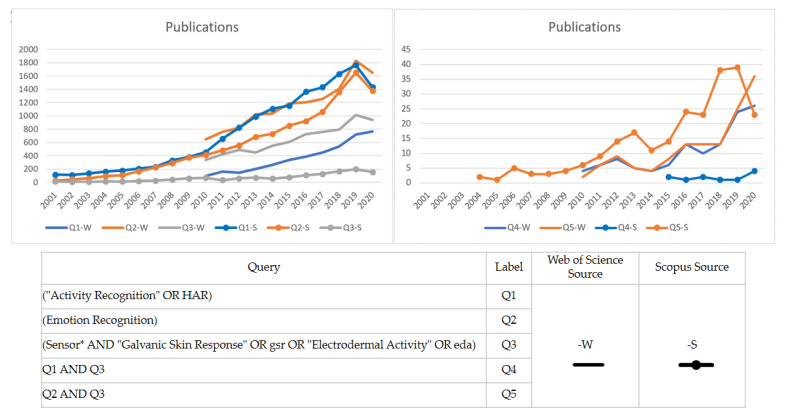
Trends in the scientific literature of human activity recognition (HAR), emotion recognition, and galvanic skin response/electrodermal activity (GSR/EDA) sensors. **Left** graph: Individual publication topics. **Right** graph: Publications involving the use of GSR/EDA in HAR or emotion recognition. *: a standard for searching queries in the scientific literature database, it helps to search finding words that start with the same letters.

Emotion recognition is a field of study historically related to sociology or psychology, understanding human behavior through techniques based on subject observation, interviews, and spoken or written expression traditionally created by experts on the matter. Nevertheless, computer science has boosted this field’s development capability by applying machine learning techniques to video or physiological data. In this way, some literature, such as [8], has proposed three essential sources of emotion detection for a smart environment: facial emotion, behavior detection, and valence/arousal detection. Nowadays, wearable devices may help valence/arousal detection, incorporating measure features such as electrocardiography (ECG), electromyography (EMG), electroencephalography (EEG), galvanic skin response (GSR), photoplethysmography (PPG), and skin temperature (ST), as per many literature reports [9,10,11,12,13]. Although multimodal measures to improve emotion recognition performance are highly recommended [11], this study focuses on evaluating the performance of GSR signals gathered from wearable sensors in different measurement places. Recent advances in machine learning techniques in the last decade and the miniaturization of hardware technologies have inspired researchers to keep working on new ways to improve human activity recognition through emotion recognition using GSR sensor technology. A quick overview of the scientific literature shows that GSR sensor technology is attracting rising interest in development and research, especially regarding activity and emotion recognition (see Figure 1). 

Many wearable devices are used to measure the GSR with various body places to measure it. Finding a suitable place to measure GSR biosignals is a valuable contribution for science and engineering to continue working on new technologies to improve quality of life, as this physiological signal is commonly used for stress-related detection [14,15]. The above impact is critical for specific populations, for example, autism spectrum disorder (ASD), to which the computer science field has contributed to this stress detection through GSR measurement for people with ASD working on computing techniques [5,16]. Moreover, other researchers are working on developing ad hoc hardware wearable devices for the ASD population. Some works for this population have proposed different measurement places, such as the waist [17], the left and right wrists [18,19], and the middle, ring, or index finger [20,21]. Nevertheless, no point of view has been established about the best place to measure GSR signals in ASD and healthy populations, although we consider it essential, for example, to measure levels of stress and to alert the people nearby or to stream information to parents or caregivers about the stress levels of people with ASD, as the literature highlights [22,23].

The above insight was our primary motivation to conduct a preliminary technical study, to determine the variability of different GSR measuring places in a healthy test group, in preparation for conducting a more extensive study with a cohort from a specific group (ASD) in subsequent phases in our research program in the Human Health Activity Lab (H2AL) [24]. The present study aimed to locate a suitable body place for measuring GSR signals among a non-homogenous population (as a real commercial device will do), using two correlation analysis workflows for four signals gathered from different places (right and left wrists, left fingers, and the inner side of the right foot). One of these workflows analyzed each signal’s correlation in the test group, while the other analyzed the correlation between the different respondents’ GSR signals. We measured each signal with the same sensor in the same place for all respondents, using video clips with pleasant and unpleasant situations as a psychophysiological activating stimulus for changes in the galvanic skin response. We used Pearson’s correlation to determine which body place is more reliable for measuring GSR in the presence of pleasant and unpleasant stimuli in a test group, RStudio as the data processing tool, and iMotion software to integrate and synchronize the data gathered by the sensors. 

The article is structured as follows: Section 2 provides an overview of the related works of correlation studies of GSR signals and sensor location. Section 3 explains the physiological signals measured and presents the sensors used and their body placements. Section 4 describes our experiment conducted and explains the method used in the present study. Section 5 presents the results from the two analysis approaches. Section 6 discusses the results and analyzes some possible outliers. Section 7 provides the conclusions and future works.

## 2. Related Work

GSR and other stress-related variables such as heart rate, and photoplethysmogram (PPG) signal are usually studied to detect emotional valence and arousal. Portable sensors for stress-related studies have hit the market, including measurement functions, resulting in studies that are cost-effective and straightforward, making popular these devices among researchers [14]. As mentioned in the introduction, the variety of affordable wearable technology devices allows measuring the galvanic skin response in different body places. Some studies have investigated the effects of GSR biosignal measurement location using wearable devices during valence changes caused by visual–auditory stimuli. Others study have investigated the measurement location or similarity and correlation measurements of affordable wearable devices compared to well-calibrated or high-quality sensors, as Table 1 shows.

Anusha et al. [25] focused on identifying the optimal configuration of dry electrodes for monitoring GSR from the wrist, as, hypothetically, electrodes designed for GSR detection are influenced by parameters such as the anatomical location of the measurement, the interelectrode distance, and the electrode material. The authors fabricated dry electrodes from stainless steel, silver, brass, and gold materials, geometrically and dimensionally similar to commercially available standard wet electrodes. They used 16 dry electrode configurations with interelectrode separations of 2 cm and 4 cm, both on the wrist’s ventral and dorsal surfaces at a 6 cm distance from the carpus. These configurations were systematically investigated, monitoring the galvanic skin response using an Analog Devices^®^ sensor unit to identify which position yielded the highest correlation with standard wet electrodes using Pearson’s correlation coefficient in order to compare GSR signals. The silver electrodes worn on the wrist’s dorsal surface with an interelectrode separation of 4 cm performed consistently well on all subjects, with an average Pearson’s correlation coefficient of *r* = 0.899 ± 0.036. 

Kushki et al. [26] conducted a correlation study between palmar and non-palmar measurement sites under cognitive and mental stressors from blood volume pressure (BVP) and electrodermal activity (EDA) signal characteristics. They measured cognitive and affective stimuli from three different body places (fingers, toes, and ear for BVP; fingers, toes, and arch of the foot for skin conductance). They evaluated these signals’ correlations using a hierarchical linear model (random effect model), gathered from a Flexcomp Infinity physiological monitoring and data acquisition unit. In this model, the dependent variable was the hand’s signal features, used as the independent and alternative sites. The results indicated a significant correlation among the GSR signal features gathered from these different body places. The cognitive and affective stimuli changes at non-palmar sites were significant from baseline (fingers), suggesting these sites for affective computing and human–machine interface measurements.

Kappeler-Setz et al. [27] envisioned a sensor system in the shoe or sock as a promising approach to long-term monitoring of GSR signals. They investigated the correlation between GSR signals measured from the feet and measurements of the fingers (most established sensing site) using an action movie as a psychophysiological activating stimulus and limb movement. GSR signals were recorded using an emotion board, attaching the electrodes to the index and middle finger’s medial phalanxes, the medial side of the foot adjacent to the plantar surface, and midway between them the first phalanx and a point beneath the ankle. The authors synchronized the two devices’ signals and calculated the Pearson’s linear correlation coefficient to compare the signals recorded from the hand and foot. This study showed changes in the galvanic skin response 88% of the time at both sensing sites, but the foot’s GSR reactivity was weaker than in hand. The authors of this study suggested foot recordings of GSR signals in daily life as a suitable location, as moderate movement has low influence on this measurement and has similar effects on both measurement sites.

Borrego et al. [28] conducted a study to compare the reliability of GSR measures from the Empatica E4 wristband against the Refa laboratory-grade system, facing emotional valence changes. The E4 is a wristband sensor with GSR electrodes located in the strap of the wristband sampling at 4 Hz in the wrist, while the Refa system consists of two Ag/AgCl sensors wired to an external amplifier sampling at 256 kHz on the fingers. Spearman’s rank correlation coefficient was used in the analysis; the α level was set at 0.05 (two-sided). The study showed low-to-moderate correlations for positive and negative images, while no significant correlation was found for neutral stimuli.

Kutt et al. [29] made a comparison of the quality of heart rate (HR) and GSR signals among four wearable devices (Microsoft Band 2, Empatica E4, Health Sensor Platform, and BITalino (r)evolution), with a professional fitness device used for HR tracking (Polar H6) as a reference. MS Band 2, eHealth, and the reference Polar H6 provide direct HR measurements. E4 and BITalino do not provide direct HR information, as they record BVP. To compare the devices, they used the Pearson’s correlation coefficient from the recorded signals. The authors suggested focusing on BITalino combined with MS Band 2 in future works. The correlation of HR signals was better between MS Band 2 and the data reference measured from Polar H6. The authors also commented that the GSR measurements of BITalino and Empatica E4 are sensitive to device placement. The correlation factors for BITalino decreased with each experiment, most likely caused by reusing ECG electrodes, as they should be replaced frequently. The authors also found a lower amplitude of skin conductance response from Empatica E4 than eHealth and BITalino, which may have been caused by different sensor locations (Empatica on the wrist and eHealth and BITalino on fingers).

Sagl et al. [30] measured the physiological variables of heart rate, and galvanic skin response, and derived heart rate variability. This was carried out simultaneously with high-quality laboratory recorder bioelectric signals (VarioPort) and two wearable devices (Zephyr BioHarness 3 and Empatica E4) while the participant was cycling on an ergometer. The study sought to demonstrate an approach for quantifying the accuracy of low-cost wearable devices compared to high-quality laboratory sensors. In the study, the authors used Pearson’s *r* correlation, the maximal information coefficient (MIC), local time series similarities, the Fréchet distance, and dynamic time warping (DTW). The authors reported lower similarities in GSR correlations due to different measurement methods, placement of the sensors on the palm of the hand vs. the wrist, the use of electrolyte gel or not for the electrodes of VarioPort (but not with the other three devices).

Phitayakorn et al. [33] conducted a study to determine the practicality of the Bandu wristwatch (manufactured by Neumitra Inc., Boston, MA, USA) to measure GSR in operating room team members during surgical simulations, wearing a sensor on the wrist and the ankle. They used Pearson’s correlation to determine the relationship between sensor data from the wrist and the ankle. The study reported a lack of a correlation between ankle and wrist sensors. The study suggested that wrist sensors are more sensitive at measuring GSR fluctuations than ankle sensors, which may be because of the anatomic variations in the eccrine gland concentration around the ankle.

Poh et al. [31] developed a wrist-worn wearable device. They conducted a study of continuous GSR measurement at both the palmar and distal forearm sites in the long-term outside of a laboratory setting, during physical, cognitive, and emotional stressors. The study found that the ventral side of the distal forearm is a viable alternative to the traditional palmar sites for GSR measurement. They calculated Pearson’s correlation coefficients and the corresponding *p*-values to measure the similarity between the GSR signals recorded from the right fingers with a Flexcomp physiological monitoring and data acquisition unit. The wrist-worn GSR sensor module developed by the authors recorded the left fingers and right distal forearm using Ag/AgCl electrodes and the left distal forearm using conductive fabric electrodes.

Kasos et al. [32] used the Obimon EDA wearable sensor for five measurement sites (fingers, feet, wrists, shoulders, and calves) to evaluate the galvanic skin response in 115 participants using emotion induction with music. The study confirmed that fingers and feet are the most responsive to stimuli. The authors also concluded that the wrist responds less to stimuli and shows a lower signal amplitude than the other two measurement sites, recommending this measure site only if the fingers feet are unavailable. Shoulders and calves are the less recommended sites by the authors.

In light of these, the literature review shows research interest in the measurement of stress-related signal location. From the Human Health and Activity Laboratory (H2AL) [24], we contribute to expanding the mass of knowledge about the galvanic skin response and wearable technologies available on the market, correlating stress-related biosignals from the right and the left forearm measured using Empatica E4 to those of the left fingers and inner side of the right foot gathered from using Shimmer3GSR. 

## 3. Physiological Parameters and Sensors

### 3.1. Galvanic Skin Response (GSR)

Also known as EDA, GSR is a measuring unit of surface resistance skin or conductivity. It can be measured by passing a microcurrent of electricity through a pair of electrodes located near one another, amplifying and registering current variation. This variation is possible as the skin resistance depends on skin humidity (sweating), the thickness of the outer layer of the skin (epidermis), and vasoconstriction, among other things [34]. Sweating behavior is sensitive to emotional stimulation due to the sweat glands being controlled by the autonomic nervous system (ANS) [35], which controls the body’s other physiological responses such as heart rate, temperature, and pupil diameter. The physiological response of the ANS can increase in the presence of stress and multiple stimuli [36]. The higher the sweat response, the higher the conductivity (µSiemens) and the lower the resistance (kOhm). This physiological response behavior links the galvanic skin response to measures of emotional valence, facing pleasant (positive valence) or unpleasant (negative valence) stimuli [28,37].

### 3.2. GSR Sensors

We used four sensors for the present study—two wearable Shimmer3GSR+ and two E4 wristbands from Empatica. Both sensors are specially designed for physiological data streaming and visualization, widely known in the scientific field of physiological data analytics. Shimmer3GSR is a wearable sensor technology that offers a variety of devices to measure different physiological parameters. Shimmer3GSR+ measures the skin’s electrical conductance using finger belt electrodes or pre-gelled electrodes placed on the foot, as per this study [38]. This sensor can record raw data on SD memory cards and the data can be downloaded via desktop software. The wristband Empatica E4 is CE medical-certified in the United States and can save the raw data in a cloud account, which plots the data every time the user connects the E4 to desktop software or a mobile app [39]. Table 2 shows the signals gathered from each sensor used for the study, their units, sample rates, and body position.

**Figure 2:**
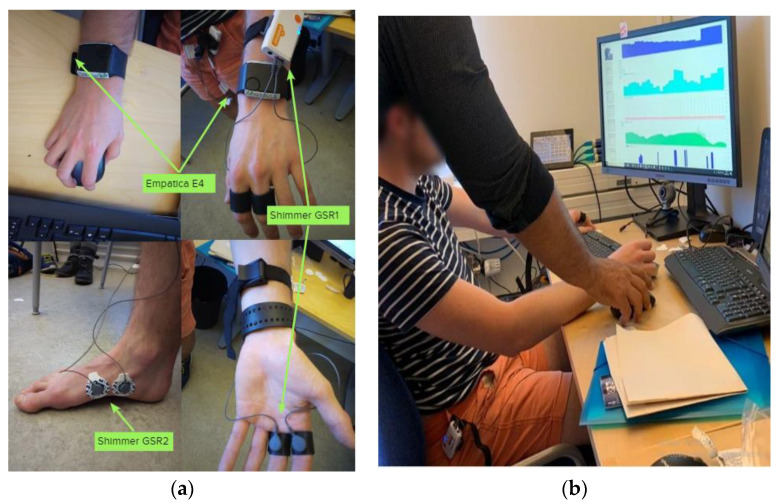
Placing the GSR sensors on a subject from the test group (**a**); checking the streaming data in iMotion software (**b**).

We measured the GSR signals from the usual sensor placing. Figure 2a shows how this sensor was placed on the study subject. Both sensors can also be paired through a Bluetooth link—this hardware feature allows synchronizing all the raw data through iMotion software. A video clip of pleasant and unpleasant sensations was configured as stimuli. iMotion software also gathered the data from the four sensors via Bluetooth, labeling the registers by signal, sensors, and time stamps each millisecond; according to each sensor’s sample rate, the register had a value or not. The advantage is that iMotion stores all raw data in one data file per respondent per study, and the timestamp marks the time series. Figure 2b shows the streaming data being gathered in iMotion software from all sensors during the setup study for one subject.

## 4. Material and Methods

### 4.1. Study Setup and Participants

Our study included 17 subjects, comprising 3 females within the age range of 30–39 years and 14 males within the age range of 23–53 years, some recruited via e-mail and others just asking to participate in an experiment. We carried out the study approved by the ethics board of the Department of Computer Science, Electrical and Space Engineering at Luleå Tekniska Universitet in Sweden. All participants provided informed written consent.

Each participant was recruited for the study at different times. We gave general instructions to the respondents, explaining the purpose of the study and how it will be conducted, asking them to read and sign the informed written consent. The areas where the sensors were to be placed were cleaned with alcohol wipes to help the sensors adhere better to the skin’s surface. After placing the sensors, the participants were asked to choose the best comfortable chair and to establish a set point of comfortability for sitting in front of a screen to avoid unnecessary movement during the experiment. Then, they were asked to put on a headset to test the sound and video; afterward, data sensor acquisition was checked, asking the participant to be calm while checking that the iMotion software was receiving all data well. It took approximately 5 min to reach this point, at which the sensors were placed to start recording the data and the stimuli were run simultaneously by the iMotion software. The staff exited the room while the subject watched the video.

The video watched by the subjects was built based on several video clips available on the web, chosen carefully by the study team to influence pleasant and unpleasant feelings to stimulate changes in valence emotions, according to Russell’s Circumplex Model of Affect [40,41]. The video clip started with a relaxing sound and video of a Caribbean beach as a pleasant stimulus, followed by a countback clip showing danger or an accident as the unpleasant stimulus, and then a tender babies or puppies video clip as another pleasant stimulus. Then, another countback appeared announcing an expectation; again, the pattern of unpleasant and pleasant stimuli was followed, as shown in Table 3. iMotion software finished recording the data when the video stimuli had finished. It was expected that the participants would manifest negative feelings as stress about the videos of crashes, babies in danger, and breaking bones, while the babies laughing and puppies would cause positive feelings as contentment and relaxation compared to the unpleasant videos. The foregoing according to a unidimensional valence approach in this experiment, to the extent that the generated emotions become positive or negative to stimulate galvanic skin responses [42,43].

### 4.2. Data Pre-Processing

**Figure 3:**
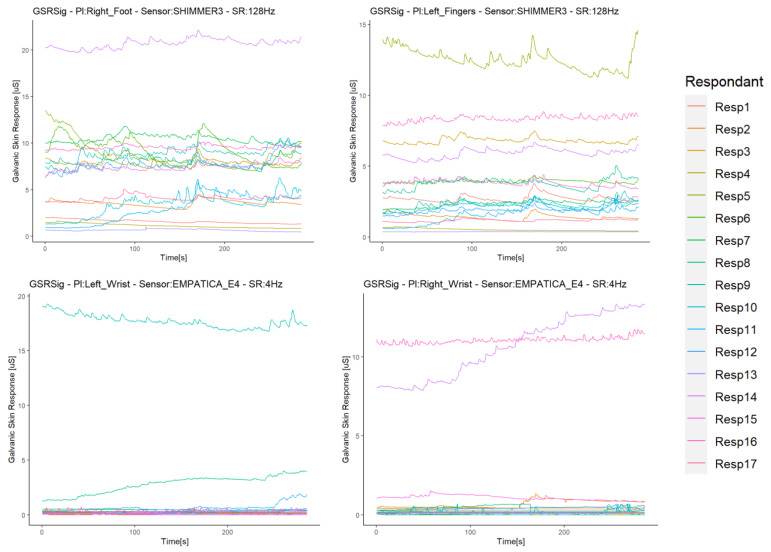
Time series of each signal dataset for the respondents.

We integrated and synchronized the data gathered by the sensors in the iMotion software. This software extracted all of the experiment’s raw data in one data file per subject with a timestamp per millisecond. The sensors streamed the data, since they synchronized with the iMotion software, but they did not link in the same milliseconds. Thus, the data started to record when the respondent selected “start” on the screen (timestamp zero), as the stimuli ran into the iMotion software. Therefore, these raw data returned by the software did not need any temporal alignment or transformation, as all sensors provided a signal in µSiemens (uS) (skin conductivity) and in the same format. The results were time series per signal with a resolution in 1 ms, in which any missing data should have been due to differences in the sampling rates. Thus, we filled these by spline interpolation, used for the correlation study of GSR signals [44,45]. For comparison purposes, the data were resampled at 250 ms, starting for the timestamp 250, as the sensors’ low sample rate was 4 Hz. As all signals were of the same type, we worked with these GSR datasets from this point, as did Sagl et al. [30]. Figure 3 shows the time series of each signal dataset for the respondents.

### 4.3. Statistical Analysis of the Signals 

We adopted two analysis approaches: one analyzing each signal’s correlation in the test group to determine the behavior of the signals in a heterogeneous test group, as any GSR signal-based stress detection system should work, the other analyzing the correlation between the signals measured in each respondent and comparing them among the test group. The literature documents that the Pearson’s correlation method fits with time series data analysis for physiological signals [46], so this was used for both workflows. We carried out all of the data processing and plots in the statistical computing software RStudio. The analysis was based on a visual examination of the Pearson’s correlation coefficient (*r*) using clustered correlograms, histograms, boxplots, and some statistical dispersions such as the mean, standard deviation, and coefficient of variation as the statistical estimates of variability in the detection of changes in the galvanic skin response among the test group with heterogeneous respondents.

## 5. Experimental Results

In this experiment, we measured GSR signals in 17 respondents with four sensors located on different parts of the body: the right and left wrists, left fingers, and the inner side of the right foot, gathered from the sensors Shimmer3GSR and Empatica E4. For each measurement location, one dataset was created, each containing the data of the signal measured (µSiemens (µS)) in the same place in 17 different respondents, and every respondent dataset had 1142 registers of GSR measures from 258 s every 250 ms (see Section 4). Every respondent watched the same pleasant and unpleasant video to stimulate changes their galvanic skin response.

**Figure 4:**
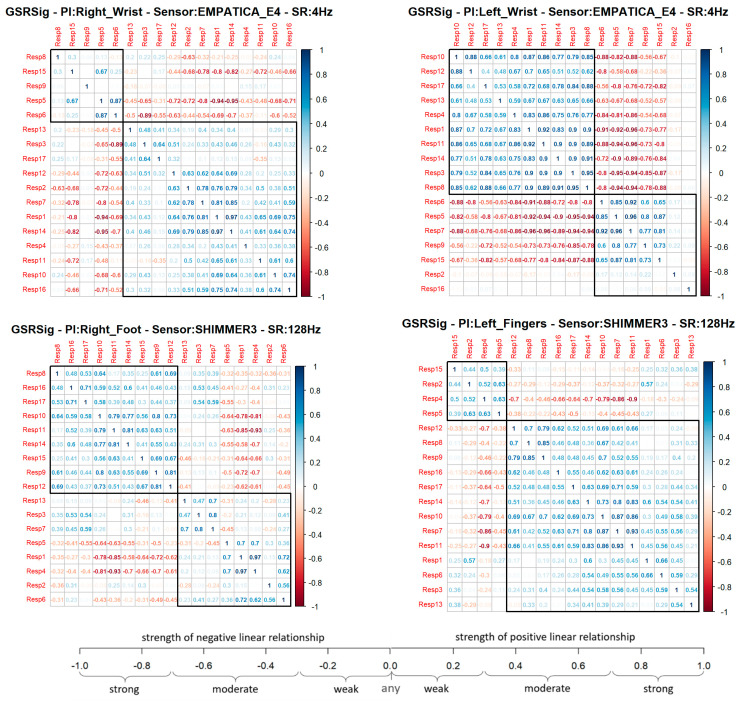
Correlation matrix of each GSR measurement location.

In the first analysis approach, the Pearson’s coefficient (*r*) provided an associative measure between two respondents to determine if the GSR measurement locations correlated with the other measurement locations or between two respondents, as well as to measure this relationship’s strength. This association indicates how many changes or variations that occurred in one respondent also occurred in the other. This association does not imply causality; instead, there could be a strong correlation between two signals and a linear response between one another, but one does not cause the other. Thus, this experimental study was based on a relational hypothesis: how strongly correlated could a GSR signal measured in specific body locations be in a heterogeneous test group? We used Pearson’s correlation to compare each signal measured between every respondent as a paired comparison, generating a correlation matrix per GSR measurement location. Each correlation matrix was plotted in a clustered correlogram using the R function hclust() for the correlation plots (see Figure 4), providing a visual image of how much a GSR signal from a particular place of measurement can be measured homogeneously in a test group, as any real stress or emotion recognition solution will work.

In the correlation matrix shown below (Figure 4), blue represents positive correlations (Pearson ‘s coefficient, *r* = 0 < 1), which means, if a respondent’s signal increased or decreased, the other tended to increase or decrease, respectively. Meanwhile, red represents negative correlations (Pearson’s coefficient, *r* = −1 < 0), which means that if, in a respondent, a signal increased, the other signal tended to decrease (and vice versa). Each cluster in the correlograms (black frame) agglomerated the respondent’s group, with more positive correlations between one another. A quick visual analysis shows that the GSR signal measured in the left fingers had a bigger group of respondents with a positive correlation, followed by the signal measured in the right wrist, left wrist, and then right foot. However, it is visually remarkable that the left wrist demonstrated the strongest correlations in the group. The bigger the group, the better the chance to detect homogeneous changes in the galvanic skin response in a heterogeneous test group from the signal measured in that place. Nevertheless, groups with strong blue correlations could mean that the matrix’s signal has a better chance of being measured with similar increasing behavior.

Each signal measured from a particular place of measurement was correlated among the 17 respondents, providing a total of 136 correlations calculated for each GSR measurement site. The difference in the number of positive correlations was not too big in the four locations—60% on average, which means the GSR measurements in the right wrist, the left wrist, the right foot, and the left fingers could have an approximately 60% probability of detecting increasing or decreasing of galvanic skin responses in a test group linearly. Nevertheless, the coefficient’s magnitude indicates how strong or weak this linear relationship of the signal is between the test groups. In order to determine this, some statistics were calculated. Table 4 shows these statistics for each positive or negative correlation of each measurement location.

We used the Anderson–Darling (A–D) and Kolmogorov–Smirnov (K–S) tests as the goodness-of-fit tests to determine if the correlations of each GSR measurement location follow a normal distribution. The positive correlations of the GSR signals measured in the right wrist, right foot, and left fingers had a normal distribution, as their *p*-values (K–S test) were higher than 0.05 of statistical significance. Statistically, if measuring a GSR signal in a test group, there is a probability of 90% of the left fingers having a positive linear correlation and a weak–moderate strength association, due to the Pearson’s coefficient being between 0.23 and 0.67; a probability of 64% of the right foot having a positive linear correlation and a weak–moderate strength association due to the Pearson’s coefficient being between 0.18 and 0.65; a probability of 53% of the right wrist having a positive linear correlation and a weak–moderate strength association due to the Pearson’s coefficient being between 0.13 and 0.62. Although these three signals had a normal distribution in their negative correlations, we did not consider them in the analysis, as some physiological factors among respondents can cause a decrease in the GSR signal while others can cause an increase, which will be discussed in the next section. 

**Figure 5:**
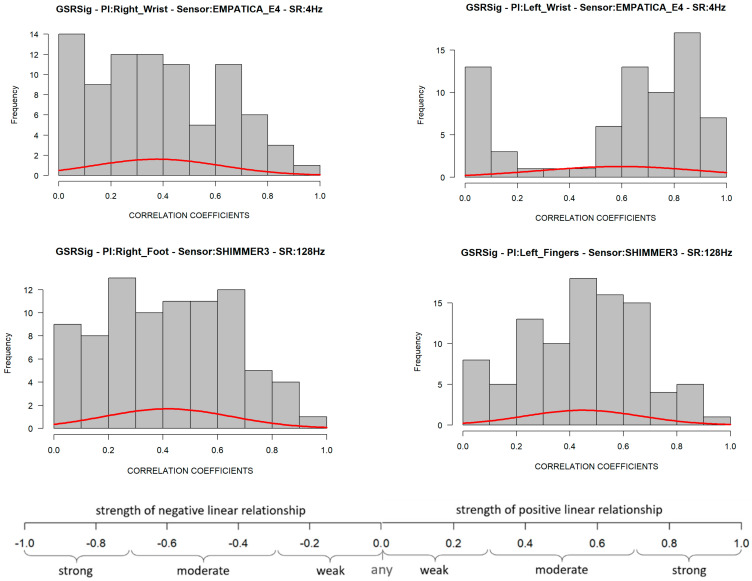
Distribution of positive correlations from the correlation matrices of each GSR signal. The bottom part is the strength of the association scale of Pearson’s correlation coefficient.

The GSR measure on the left wrist also failed the goodness-of-fit test in its negative and positives correlations. Its *p*-value was less than 0.05 (statistical significance), which means that these signals’ positive linear relationship was not uniform in the test group. This measurement location has a very disperse correlation among the test group, so we could not achieve statistical inference as we did for the other three GSR measuring sites. A visual inspection to check the symmetry of the positive correlations of each signal (Figure 5) allowed us to observe that, unlike the GSR signals from the right wrist, right foot, and left fingers, the signal from the left wrist had a remarkable asymmetric distribution in its correlations among the test group. Several correlations fit the different correlation scales, which implies non-homogeneous behavior in this GSR measurement location.

**Figure 6:**
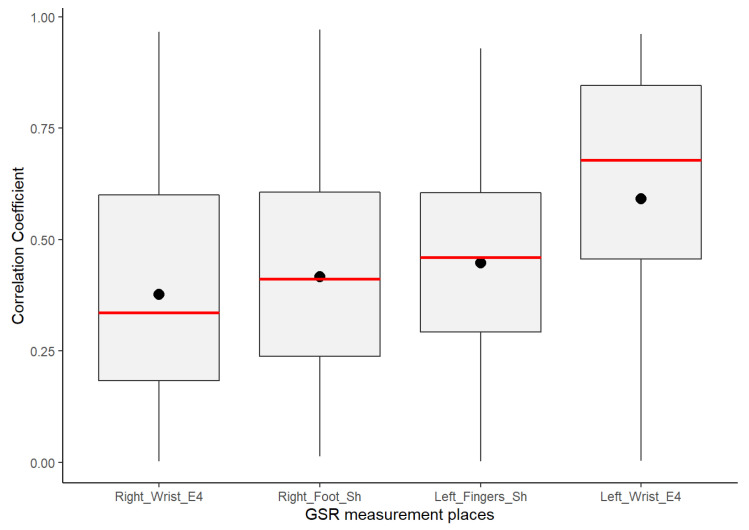
Box plot distributions of the positive coefficient correlations from the correlation matrices of each GSR signal.

Despite GSR signals from the right wrist, right foot, and left fingers all having weak–moderate positive correlations, the mean of the positive correlations of each measurement location provided a better idea about which could have better reliability for detecting increasing or decreasing of galvanic skin responses homogeneously in a test group. As shown in the box plots in Figure 6, the signals from the left fingers and the right foot have better reliability than the other signals, as the mean (black point) and median (the line that divides the box into two parts) are close and both have certain symmetry in the standard deviation. The GSR signal from the left fingers followed by the right foot have better skewness, which means that there is a better probability that measurements of GSR in these sites have a moderate linear positive relationship in the test group. The signals measured from the right wrist have a more extensive dispersion than the two mentioned above; the mean and median tend toward a low–moderate positive correlation in the test group.

From Figure 6 above, despite the whiskers of each signal denoting dispersion of positives correlations in all of the strength association scales, the statistical analysis did not indicate any positive correlation outlier; the coefficient of variation (CV in Table 4) offers a better understanding of which signals have a lower tendency of dispersion in their positive correlations. Less dispersion means a better chance that the GSR measurements in a test group fit a specific probability correlation of the strong correlation scale. The lower the coefficient of variation, the less the dispersion, the better the chance than if the GSR signal increases, will do in another respondent simultaneously in a particular association strength interval. The statistics from Table 4 show that the lowest correlation dispersion in the test group is in the signals from the left fingers (CV = 0.49), followed by the right foot (CV = 0.56) and then the right wrist (CV = 0.65). The above has a significant effect on reliability, providing some insight between these four measurement locations. Despite the above, even based on Figure 6, the right wrist’s GSR signal cannot be considered the strongest positive linear relationship between the four measurement sites. As mentioned above, it has an asymmetric distribution in its positive correlations (Figure 5), and it does not meet a normal distribution (Table 4; the *p*-value is less than 0.05). 

**Figure 7:**
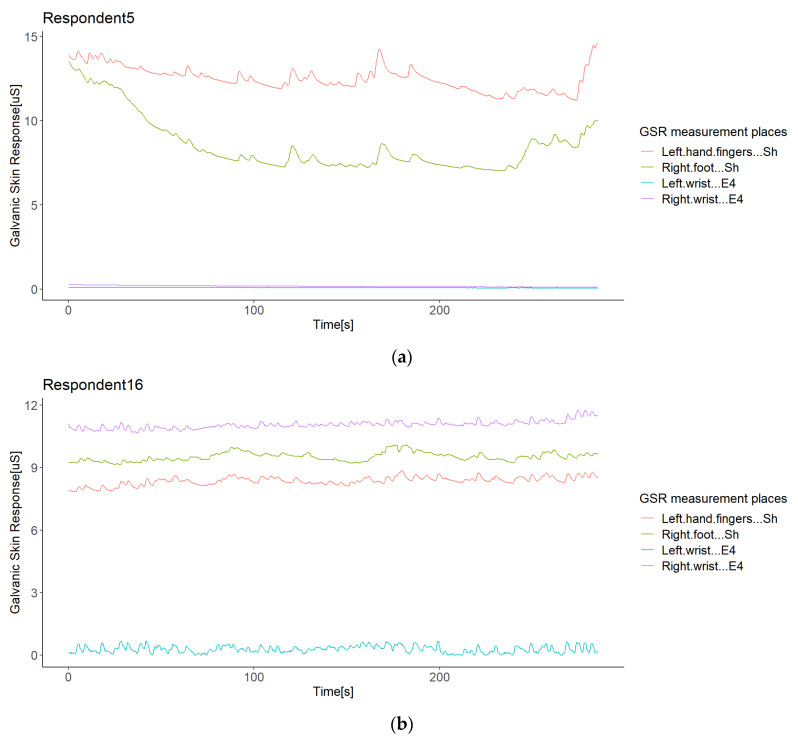
Illustration of the differences in the GSR measurement range observed in the experiment: a case of differences between two types of sensors (**a**); a case of differences in different types of sensors (**b**).

Although the statistical analysis pointed out no positive correlation outliers, possible sources of correlation variability were inquired in order to see which errors or bias in the sampled data of each respondent may lead to a non-homogeneous response of the galvanic skin response in the test group in a particular measurement location, especially in the signal from the left wrist. We conducted a visual inspection of the signal time plots of each respondent. To illustrate the findings, we only show some representative graphics from the experiment. The first impression was that the GSR signals from the left and right wrists registered relatively small values compared to the GSR signals from the right foot and left fingers. The first two signals were measured with Empatica E4 sensors, while the other two with Shimmer3GSR sensors (Figure 7a), so it is remarkable that these sensors tended to measure in a range of µSiemens (uS) higher than Empatica E4 (by 3–5 in most cases). Moreover, 89% of the time, the measurements from the right foot were higher than from the left fingers (Table 5).

**Figure 8:**
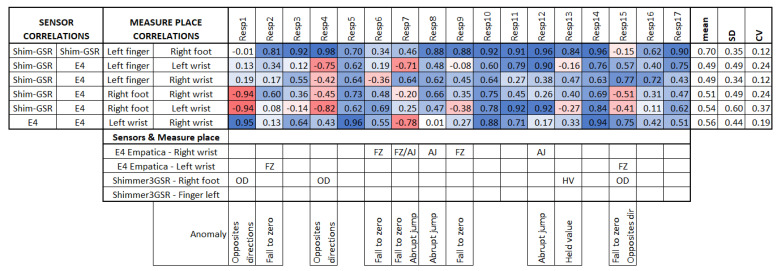
Correlation between signals in each respondent. In the bottom part, the anomalies detected relate to each respondent, measurement location, and sensor. OD, opposite directions; FZ, fall to zero; AJ, abrupt jump; HV, held value.

In order to see if differences in the GSR magnitude readings could affect the detection of homogeneous increases or decreases of galvanic skin responses in the test group in specific measurement sites (as well as other possible anomalies), we used the second analysis approach based on the correlation between the signal measurement sites in each respondent, and compared them among the test group. Figure 8 summarizes the linear correlations among the sensors of each respondent, both positive and negative, as in this case, the comparison was between signals of the same respondent, and no factors should affect the measurement in specific places but the respondent themselves or the sensor used, which is of interest in this study. 

Empatica E4 sensors have a lower range of measurement than Shimmer3GSR sensors (Figure 7a); this was the case in 82% of the test group. However, in the other 18% of the test group, one of the Empatica E4 sensors read a higher magnitude than the other Empatica E4 sensor (see Figure 7b). From the left wrist of Respondent 9 and the right wrist of Respondents 14 and 16, the measurements of one of the Empatica E4 sensors were higher-magnitude signals than the other E4 sensor signals. Then, to investigate whether the magnitude of the measurements influences a better correlation among the test group, we conducted a paired comparison between the correlations involving these signals with other respondent anomalies (Figure 8), indicating no significant difference. Thus, we cannot affirm that higher magnitude measurements cause more homogeneous readings among a test group, but we can affirm the type of sensor. 

Based on the insight and previous results from the first correlation analysis approach, a quick horizontal view in Figure 8 highlights that the signals measured from the right foot and left fingers still demonstrate a homogeneous response in the test group, as they have a higher correlation mean, a lower standard deviation, and a lower coefficient of variation. This means that these two places of measurement had a strong linear response in the test group, meaning that—independent of any factor from a heterogeneous group—in both places, if the galvanic skin response increases or decreases, the other measurement location will tend to increase or decrease with a strong association. This association is about how many changes or variations co-occur in one respondent and will occur in the other.

**Figure 9:**
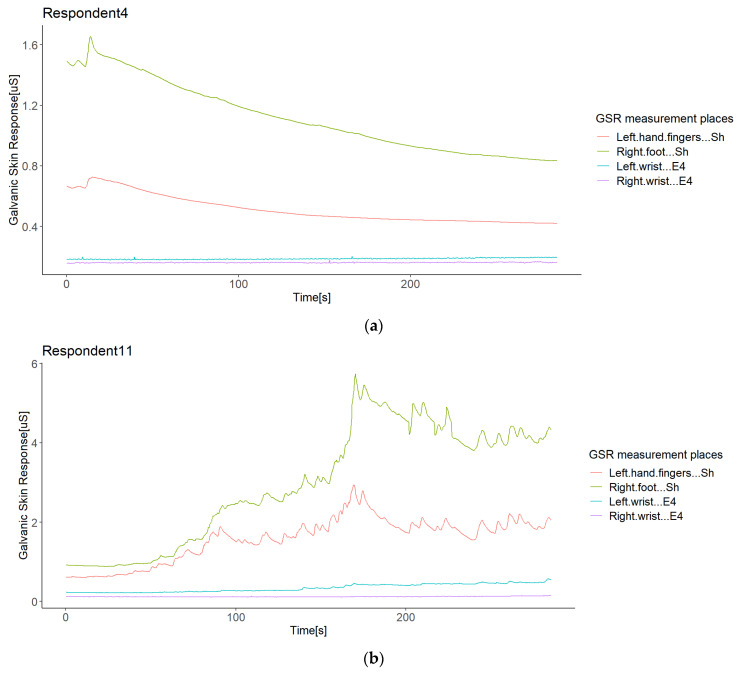
Illustration of the opposite directions of GSR signals in the same respondent (Respondent 4). A case of different types of sensors: A respondent with negative correlations between different sensors (**a**); a respondent with positive correlations between sensors (**b**).

From the second correlation analysis approach (Figure 8 above), it can be appreciated that not all respondents followed a positive linear relationship in the GSR signals measured from their bodies (Figure 8), which means that in a respondent, not all GSR measurement sites increased when the others increased. We found two different cases for this negative correlation between sensors or measurement sites in the same respondent (from now, termed opposite directions). In the first case of this opposite direction anomaly, some respondents experienced increased signals from some GSR measurement sites using one type of sensor, while the others decreased using another type of sensor—for instance, Respondent 4 (Figure 9a). Although the increasing signals of the Empatica E4 sensors cannot be fully appreciated, the negative correlations between these two types of sensors in Respondent 4 denote this (Figure 8). In this case, signals from both the right foot and left fingers (from the Shimmer3GSR sensors) decreased in an almost perfect positive correlation (*r* = 0.98; see Figure 8), while the signals from the left and right wrists (from the Empatica E4 sensors) increased with a moderately positive linear relationship (*r* = 0.43; see Figure 8). 

**Figure 10:**
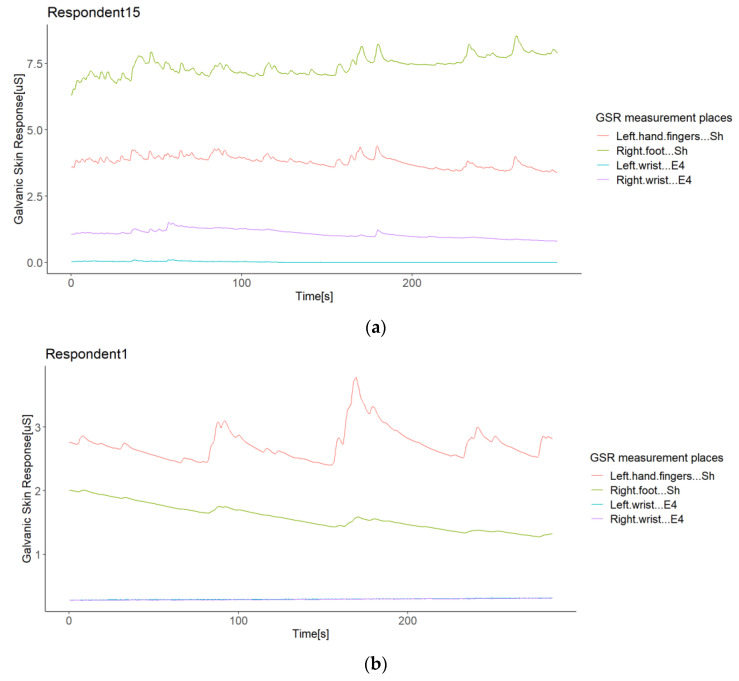
Illustration of the opposite directions of GSR signals in the same respondent (Respondent 15). A case of the same type of sensor: A respondent with negative correlations between different sensors (**a**); a respondent with positive correlations between sensors (**b**).

The second case refers to Respondents 15 and 1, where the correlation between Shimmer3GSR sensors was negative (Figure 8). When a sensor measure increased, the signal from the other same type of sensor decreased, as Figure 10 shows. Despite the Shimmer3GSR being related in both cases, the signal of the right foot presented an opposite behavior; this is easy to infer, as is this is the only signal measured with the Shimmer3GSR sensors to present a negative correlation with both Empatica E4 sensors. Plus, a visual examination of the time signals plot of each respondent involved (Figure 10) indicates that this second case reported no increasing or decreasing tendency, as the signal increased in one respondent while it decreased in others. For this case, we did not consider the negative correlation between the Empatica E4 sensors in Respondent 7, as these correlations demonstrated some anomalies in the signal, which will be analyzed next.

Besides the above anomaly, we highlight other anomalous findings in the GSR signals measured during the experiment: sudden falls to zero and abrupt jumps of magnitude, present among the measurements made with Empatica E4. Only one measurement with one of the Shimmer3GSR sensors presented a signal with values that remained consistent for a relatively long time. The bottom of Figure 8 relates the respondent, measurement location, type of sensor, and the respective correlation anomalies. This type of error comes from the sensors and their use on the respondent, especially the place of measurement. From the correlations in Figure 8, the first impression is that the Empatica E4 sensors have a higher rate of measure anomalies than the Shimmer3GSR sensors. The second impression is that the fall to zero (FZ) anomalies are the most frequent type of error in the Empatica E4 sensors. First, this could have been because the battery level may have been low during the experiment, but checking the raw data generated by the iMotion software from both Empatica E4 sensors, in which the battery level was also captured, no patterns related to the anomalies were observed. 

**Figure 11:**
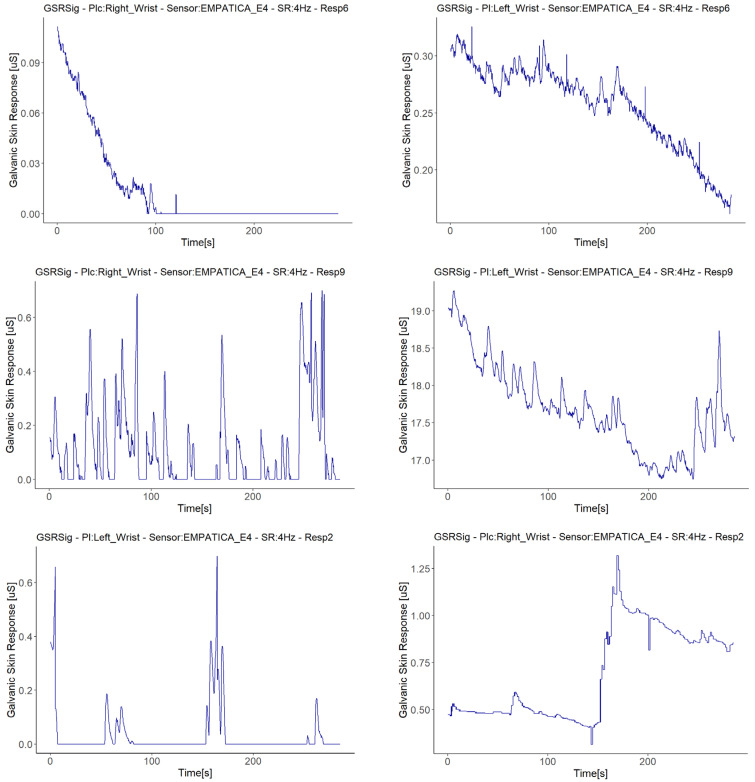

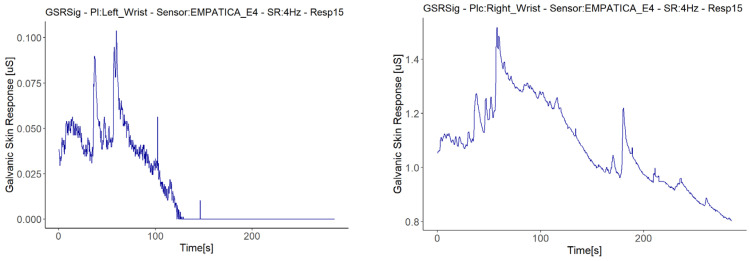
Illustration of cases with fall to zero anomalies detected. On the **left** are the signals with anomalies, while on the **right** are the other signals measured in the same respondent with the same sensor type.

Four of the five signals with fall to zero anomalies had a positive linear relationship with the same type of sensor (Empatica E4): Two of these demonstrated weak strength correlations, while the other two achieved moderate-strength correlations, despite these signals having a zero value for almost half of the measuring time. Moreover, a visual examination provided evidence that this anomaly occurred when GSR signal decreased (see Figure 11), as all of the signals from the Empatica E4 sensors showed decreasing GSR values in different respondents. The above, plus the fact that the Empatica E4 sensors showed a lower range of GSR measurements during this experiment, we can say that if the GSR measurement range is below 0.6 µS from an Empatica E4 sensor, then the decreasing trends of the galvanic skin response might result in this signal falling to zero suddenly, unlike other sensors. This may not be due to the measurement location, but rather the type of sensor, as the left wrist measurement location also had a fall to zero anomaly, but with a different Empatica E4 sensor. Additionally, it cannot be said with certainty that the anomaly of fall to zero influences low correlations, as the signals with this anomaly measured with one of the Empatica E4 sensors had higher or positive correlations than the other E4 sensor, which did not present anomalies.

**Figure 12:**
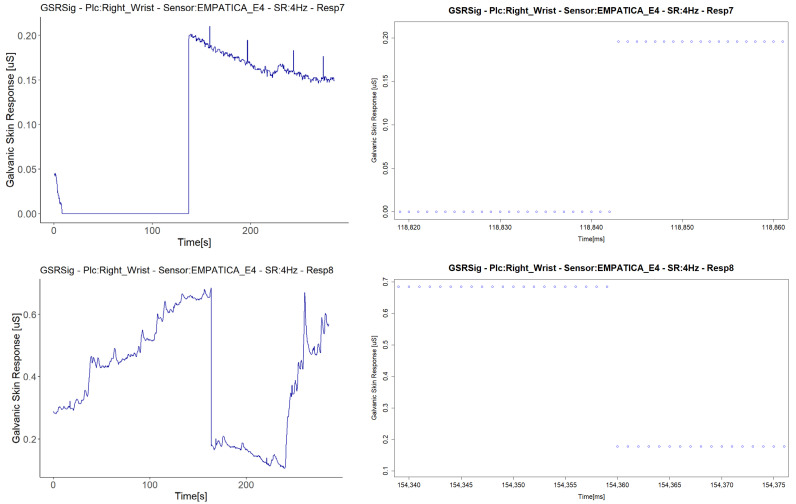

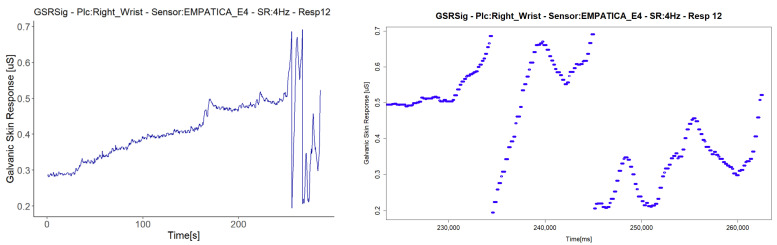
Illustration of cases of abrupt jump anomalies. On the **left** are the signals with the anomaly turned into a graph from the downsampled data; on the **right** are the other signals measured in the same respondent with the same type of sensor, turned into a graph from the raw data, zooming in on the time interval.

We treated the fifth signal with fall to zero anomalies separately, as this is the only signal with this anomaly that presented a second anomaly in the same sensor (E4 in Respondent 7). We attribute the negative correlations in this respondent to a second anomaly in the signal, an abrupt jump (AJ) (see Figure 12), as the correlations with abrupt jump anomalies are those that presented lower correlations between Empatica E4 sensors (Figure 8). Nevertheless, this evidence is not enough to affirm that this anomaly may affect homogeneous change detection among the test group. There were correlations between signals both with and without abrupt jump anomalies it, with better correlations for those with than without any anomalies in their signals (for instance, Respondent 7 vs. Respondent 11). Neither can we attribute these abrupt changes to fast changes in the GSR measure, as this physiological parameter does not behave in this way. What happens is a sudden change in magnitude, as can be seen in the right-hand plots of Figure 12, where every dot is a GSR value measured in milliseconds, that, unlike the left plots that came from the dataset sample rated in 250 ms, came from the raw data.

**Figure 13:**
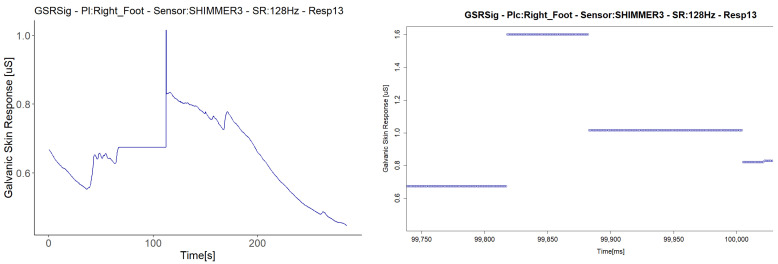
Illustration of the detected constant value anomaly. On the **left** are the signals with the anomaly turned into a graph from the downsampled data; on the **right** are other signals measured in the same respondent with the same type of sensor, turned into a graph from the raw data, zoomed in on the time interval.

The last anomaly detected with the Shimmer3GSR sensors was a sustained measurement value for a relatively long period (Figure 13). The signal in Respondent 13 remained constant at 0.67 µS for 120 s and then registered an abrupt change in magnitude held at a specific value for several milliseconds. Despite this, the anomaly in the signal seems not to have affected the homogeneous detection of increasing or decreasing the galvanic skin response in the test group. Figure 8 shows a really strong, almost perfect linear positive correlation between the pair of Shimmer3GSR sensors.

**Figure 14:**
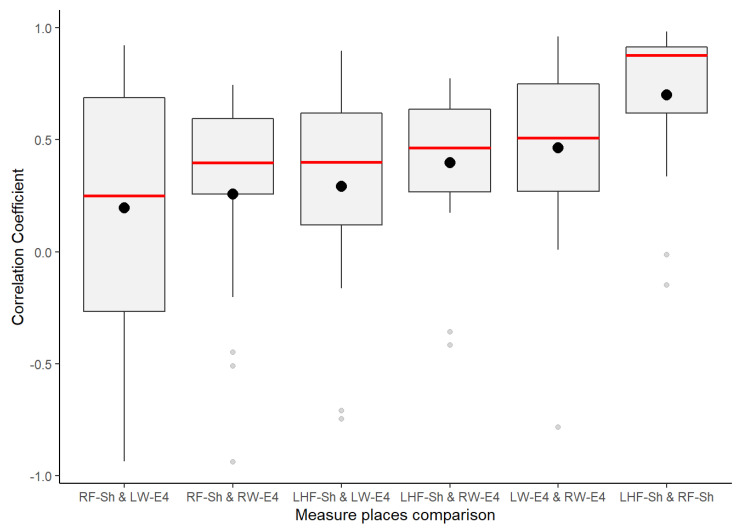
Box plot distributions of the coefficient correlations from the correlations between signals.

Finally, this second correlation approach analysis highlights some insights from the statistics in Figure 14. Better homogeneous detection of the test group in this experiment was present in the signals from the left fingers and right foot, both measured from the Shimmer3GSR sensors. The statistics show that the second best mean correlations came from the right and left wrists measured using the Empatica E4 sensors. From the above, we can affirm that GSR signals changes can be measured homogeneously in a test group, at least in two different sites, with a strong positive linear correlation when using the same type of sensor (at least Shimme3GSR and Empatica E4). Compared to the above, as correlations of measurement location that involve different sensors have, in general, a moderate positive correlation strength, we can affirm—for this experiment—that the type of sensor does not influence, in a homogenous detection of GSR, change among a test group, Shimmer3GSR being the sensor that provides better results. 

The outliers in Figure 14 may be due to the anomalies previously discussed, which caused negative correlations between the sensors in each respondent, especially in the right foot and left wrist correlations, which may be due to anomalies in both measurement sites, as well as the high dispersion detected in the first correlation analysis approach. This dispersion may have resulted in the low strength of association for the correlation of the signal from the left fingers. A comparison between those respondents with anomalies in their GSR signals and those without indicated a particular effect on the positive correlations between signals. However, a visual inspection of the respondents’ correlation matrix of each signal (Figure 4) against the anomalies in Figure 8 evidences some cases in which the anomalies did affect the correlation between respondents, and other cases not affected, as low or negative correlations between respondents without anomalies in their signals. 

## 6. Discussion and Limitations

Using Pearson’s correlation coefficient (*r*), we compared the signals measured from four wearable GSR sensors used on the right and left wrists, the fingers left, and the right foot to investigate which place of measurement could have a better homogenous response in a test group. Our experimental results indicated that these four GSR measurement locations have a significant tendency toward a moderate–strong positive correlation—on average, a 60% probability of detecting linear increasing or decreasing GSR changes in a test group (Table 4). The GSR signals measured in the left fingers achieved a bigger group of positives correlations, followed by the right foot, the right wrist, and then the left wrist (Figure 4). Statistically, there is a 90% probability that the GSR signals measured in the test group would have a positive weak–moderate strength association if the measure is in the left fingers, 64% if in the right foot, and 53% if in the right wrist. The GSR signals from the left wrist failed the goodness-of-fit test in the negative as in the positives correlations, and we were unable to make statistical inferences.

The GSR measurement locations of the left fingers and right foot showed better reliability for a moderate–strong correlation among a heterogeneous test group than the other measurement sites, as both had certain symmetry in their standard deviations and had better skewness in their correlations (Figure 6). This provides an approach to determine how many changes or variations occur in one respondent and will occur in another. Analyzing the correlations between signals, on average, the left finger and right foot GSR measurements showed the strongest positive linear responses between the signals in each respondent (Figure 8). This means that, independent of any factor in a heterogeneous group, in both places if one signal increases or decreases in terms of their galvanic skin response, the other measurement locations will tend to increase or decrease, respectively, with a strong association (Figure 14). Thus, from this experiment we can infer that there is a better probability to detect homogeneous changes in the galvanic skin response of a heterogeneous test group when the GSR measurements come from the left fingers or the right foot, consistent with the findings of [27,32,47]. The signals measured from the right wrist had a more significant dispersion of positive correlations in the strength association scale and had the lowest means and medians of its positives correlations in the test group, tending toward a low–moderate strength (Figure 6), also consistent with previous findings [47,48].

Insights from these experimental results for highly homogeneous detection of changes in the galvanic skin response of a test group came from the left wrist. Despite the positive linear relationship of this GSR measurement location not being uniform in the test group, its correlations fit in the high as well as the low strengths of positive association and have the highest means of negative correlations. It is visually remarkable that the left wrist had the strongest positive correlations from the signal matrix correlations (Figure 5), as well as the highest means (Figure 6). The hidden potential could stand out, inferring that this signal could have a potential high-strength correlated GSR measurement location among the test group without any outliers affecting the correlation. Anyhow, specific studies should be conducted to determine if the influence on the Empatica E4 sensor or the galvanic skin response of the left wrist is caused by internal variables such as age, gender, and culture or external variables such as temperature, relative humidity, clothing, or medication according to Boucsein et al. [49].

This study also showed a weak and negative correlation between the signals in some respondents. Looking for possible outliers, we reported anomalies in GSR signal measurements as sudden falls to zero, abrupt jumps of magnitude, opposite directions of GSR signals in the same respondent, and held values in a particular interval of time. We found the opposite direction anomaly in two cases. In the first case, a respondent had GSR signals measured from different body sites, one signal increasing using one type of sensor while decreasing using another type of sensor (Figure 9). In the other case, the GSR signals went in opposite directions measured with the same type of sensor (Figure 10). 

This opposite direction anomaly is distinguishable in the right foot measured with a specific Shimmer3GSR sensor (Figure 8). This issue was also reported by Borrego et al. [28], who compared the reliability of two GSR wristbands and found that while the Empatica E4 detected an average increase in the GSR for unpleasant stimuli, the Refa system registered the opposite tendency. The authors gave credit to the Refa system, as previous studies have shown the Refa system to follow a good tendency for unpleasant stimuli. This may be in accordance with the statement that the ankle may be less sensitive to GSR fluctuation according to Phitayakorn et al. [33] and Betancourt et al. [22]. The first author found no correlations between the GSR signals measured from the wrist and the ankle (a place close to the inner side of the right foot used in this experiment). Moreover, this decreasing GSR values may be to the lower skin temperatures of the right causing the permeability of the skin to water to decrease [26,49]. In this opposite direction anomaly, we did not find any tendency to increase or decrease, as the signal of the right foot with the opposite direction anomaly increasing in one respondent while it decreased in another.

A look in depth at the weak or negative correlations in the same respondent lay in the recent theory proposed by Picard et al. [50] about bilateral differences and asymmetric GSR measures mainly present in the two halves of the upper body. As this theory gains more and more strength in the EDA research field [32,51,52], and the negative correlation found in this study came from two halves (the upper and the bottom parts of the body), more specialized correlation studies should be conducted for asymmetric measurements in the right foot. Due to this statement, we highly recommend that from now on, new emotion detection algorithms take into account bilateral measurements to sort biases in the detection assessment among a test group.

Other data anomalies may have caused the weak or negative correlations in this study also, and it is essential to evaluate these issues in GSR measurements further, as this is not beneficial for new stress detection systems. Only the Empatica E4 sensors presented fall to zero anomalies (Figure 11). We observed that if the GSR measurement range was below 0.6 µS for Empatica E4 sensors, the decreasing trends in the galvanic skin response might cause these signals to fall to zero suddenly, unlike Shimmer3GSR sensors, which tend to be measured in a range of µSiemens (uS) higher than Empatica E4 sensors (3–5 higher in most cases, as can be seen in Figure 3). The above is in accordance with Kutt et al. [29], who reported that the amplitude of skin conductance responses from Empatica E4 is lower than from other devices such as eHealth and BITalino. The above is also in accordance with [53], who reported that an EDA measurement under high skin resistance conditions may outline low values of skin conductance, in which case slow decreases in GSR levels can reach the point where the sensor is not able to obtain a measurement, and thus a zero value is returned constantly. Even though skin dryness or hydration levels may be a factor affecting the detection of skin conductance levels, we cannot attribute this anomaly to the measurement location. However, evidence points to the Empatica E4 sensor measure levels, as this happened for two different devices in this experiment and has also been reported in the literature. Data loss in transmission was discarded as well, as the other variables collected from the E4 sensors, such as inter-beat interval (IBI), BVP, and HR, continued to transmit, in addition to a faulty connection with the electrodes due to the decreasing tendency of the GSR values. In each respondent plot, we also observed that 89% of the time, using the Shimmer3GSR sensors, the right foot signals were higher than the left finger signals.

Some sudden abrupt jumps in the measured magnitude were present in the different respondent’s signals, but the evidence is insufficient to affirm that this anomaly may affect homogeneous change detection among a test group and cause negative correlations. Neither can we attribute these abrupt changes to fast changes in GSR measure, as this physiological parameter does not behave in this way. What may happen is a sudden change in magnitude from one millisecond to another, as shown in the right plots of Figure 7. This issue was reported by Kutt et al. [29] with Empatica E4 and MS Band. During movements, the contact between the body and the sensor was not constant, leading to sudden conductance changes, resulting in lower signal correlation.

Unlike the previous cases, which occurred three times each in the different respondents throughout the experiment, we detected the held value anomaly only once. During the measuring, the signal start suddenly sustained a value of 0.67 µS without any change across 120 s (Figure 13). We visually inspected the plots generated from the pretreated dataset, and the held values were registered in the raw data to be highly likely due to the loss of connection in wireless communication. Despite this, the anomaly in the signal seems not to have affected the homogeneous detection of increasing or decreasing GSR measures in the test group.

In this experiment, the Empatica E4 sensors registered a higher rate of anomalies than the Shimmer3GSR sensors. Moreover, the fall to zero anomaly was the most frequent error in the Empatica E4 sensors. The battery level captured in the raw data in the iMotion software did not show any pattern related to anomalies. We encourage new GSR-based emotion detection proposals to consider if these anomalies and their possible causes as discussed herein are present in their accuracy assessment for the possible improvement of outcomes. The above is the reason why we did not remove the outliers for this study, as we wanted to observe the behavior of the GSR signals among the test group, as would be the case in real life. Additionally, we wanted to indirectly determine how any outliers in the raw data captured may influence the detection of increasing or decreasing GSR measures in the test group, without any computation technique applied but the reconstruction of missing data. Even as an experimental study, we state that these inferences from the results should not be taken as fact in the acquisition of physiological GSR signals. More detailed studies about these anomalies, their causes, and their consequences must be studied more in-depth, according to the measurement devices, as stated in the conclusion and future works.

Despite other studies attributing low correlations to differences in the sample rate between devices [28], our results showed moderate positive correlations between sensors with a significant difference in the sample rate (128 Hz for Shimme3GSR and 4 Hz for Empatica E4) after applying spline interpolation and downsampling the dataset to 4 Hz. In this case, due to the downsampled data and difference in timestamp synchronization, we use using spline interpolation [30].

The use of iMotion software to integrate and synchronize the data gathered by the sensors was valuable. However, our study was not without limitations. Different wearable sensors could cause data biases and could affect the correlation results, but using different models of sensors for the comparison of correlations seems to be accepted in the literature, as shown in Table 1, possibly due to the nature of the Pearson’s coefficient calculation itself. Another limitation is not using a rigorous assessment of a bidimensional approach for both the participants’ valence as well the intensity of the emotion referred to as arousal [42,43] during the experiment, as a factor of respondents’ sensitivity to pleasant or unpleasant stimuli to see how these can affect the correlation analysis among a test group. Moreover, the local skin condition may influence the reliability of the measurements and the quality of the signals. These have to be included in an in-depth correlation study looking for potential affecting factors such as different measurement methods (e.g., sticky electrodes vs. plate electrode) [30], sensor movement [26], skin temperature, skin thickness, water content, body posture, and the density of sweat glands [26,54]. It is essential to complement this study with in-depth correlation analyses using datasets of GSR signals from all respondents for each pleasant or unpleasant stimulus. As well as comparing correlations using the different techniques of smoothing and low/high filters and downsampled raw data, according to [30], low-cost wearable sensors tend to produce datasets with reduced data quality, and the noise in GSR signals gathered from wearable devices may vary [29].

## 7. Conclusions and Future Works

This paper contributes to the literature by reporting results of an introductory study of the correlations of measurement places of GSR signals with wearable sensors in a test group, relating information about possible causes of negative correlations and comparing them with some other existing findings. Although there have been a few related studies, there is still a need to find and prove correlations among different measuring places and to compare them with other studies of negative correlation issues. Seventeen respondents participated in this experiment, in which four GSR sensors were used simultaneously while watching a video clip that contained pleasant and unpleasant scenes for 285 s to simulate changes in the galvanic skin response. The experiment had a two-correlation analysis approach; one analyzed the correlation between each signal of the 17 respondents, while the other analyzed the correlation between the signals of each respondent. Both approaches used statistical estimates and visual inspection of time plots to gain insights into the homogeneity of the detection of the galvanic skin response in the test group.

The experimental results confirm previous findings that a better GSR measurement location for homogeneous detection in a heterogeneous test group may be the left fingers and the right foot—both measured using Shimmer3GSR sensors in this case. The statistics show that the second best correlation may be from the right and left wrists, also measured using Empatica E4 sensors. In general, based on this experiment, we reported that galvanic skin response changes may be measured homogeneously in a test group, at least in two body sites, with a strong positive linear correlation when using the same type of sensor. The type of sensor may not influence the homogenous detection of galvanic skin response changes among a test group, but there are some sensors such as Shimmer3GSR that may provide better results. 

Our analysis indirectly showed that Shimmer3GSR sensors may present better reliability of homogenous detection of galvanic skin response changes, as they have fewer anomalies among respondents. However, attention should be paid to the difference in the sense of the GSR signal, as in this experiment one respondent showed increasing GSR signals from the right foot while the rest of the signals tended to decrease. Our results also showed that the correlations between sensors with significant differences in the sampling rate (128 Hz for Shimme3GSR and 4 Hz for Empatica E4) were moderately positive, at least after applying spline interpolation and downsampling the dataset to 4 Hz.

Our experiment also showed that the inner side of the right foot may be suitable for measuring GSR, as it is strongly correlated with GSR measurements from the left fingers, at least when using the same type of GSR sensor. The right foot was also positively correlated, with a moderate strength of association, with the rest of the measurement sites. Regardless of the type of sensor, the magnitude of the measurement did not influence the correlations between the test groups. We did not find strong evidence that the signal anomalies detected in this study resulted in low correlations. However, they were indirectly investigated in this study. Our results underline the importance of bilateral GSR measurements for correlation studies and the accurate testing of emotion detection algorithms, as we found asymmetric measurements in different parts of the body in the same respondents. We recommend considering this condition to evaluate the accuracy of emotion recognition methods, performing bilateral measurements.

Our future works will further evaluate, in a second stage, the influence of negative correlation factors in test groups such as sensors movements, local skin conditions, type of electrode, respondent sensitivity factor to pleasant or unpleasant stimuli, technical specifications of wearable sensors, quality of signals, smoothing and interpolation methods, and internal and external factors of the GSR measurement. Moreover, we will consider bilateral body measurements due to the multiple arousal theory. Thus, robust methods will be used to describe the variation in signal correlations in the test group as the design of experiment (DOE) to understand the further behavior of signals from different measurement sites. As well as rigorous experiments assessing valence and arousal changes with facial emotion recognition systems, for a bidirectional approach Russell’s circumflex model of affect can be used. The third stage of our research program will be focused on finding a suitable wearable technology to conduct quality measures in the foot; we expect to conduct a study under conditions of movement and stillness toward a stress measurement concept technology for people with ASD. We may need to consider a DOE using two different types of sensor in each foot to evaluate which have better reliability for other populations than heterogeneous test groups.

## Figures and Tables

**Table 1:** A summary of the recent literature regarding GSR measurement places and physiological sensors.

Research Work	Focus of Research	Devices Used	Position Measured	Comparison Method
Anusha et al. [25]	Optimal dry electrode location for GSR measurement	Analog Devices^®^: Ag/AgCl, stainless steel, silver, brass, and gold electrodes	Ventral and dorsal surfaces of the wrist	Pearson’s correlation coefficient
Kushky et al. [26]	Correlation between palmar and non-palmar GSR measurement sites	Flexcomp Infiniti physiological monitoring and data acquisition unit	Fingers, toes, and arch of the foot	Hierarchical linear model (random effect model)
Kappeler-Setz et al. [27]	Correlation between GSR measurements of the feet and fingers	Emotion Board	Index and middle finger and the inner side of the foot	Pearson’s correlation coefficient
Borrego et al. [28]	Reliability measures of the galvanic skin response of a wristband against laboratory-grade equipment	Empatica E4 and Refa System	Wrist and fingers	Spearman’s rank correlation coefficient
Kutt et al. [29]	Comparison of heart rate and GSR quality signals among wearable devices	Microsoft Band 2, Empatica E4, Health Sensor Platform, BITalino, and a Polar H6 as a reference	Wrist and fingers	Pearson’s correlation coefficient
Sagl et al. [30]	Quantifying the accuracy of low-cost wearable devices in comparison to high-quality laboratory sensors	Wearable Zephyr BioHarness 3, Empatica E4, and VarioPort laboratory recorder bioelectric signals	Hand palm vs. wrist	Pearson’s *r* correlation, Maximal information coefficient (MIC), local time series similarities, Fréchet distance, and dynamic time warping (DTW)
Poh et al. [31]	Studying continuous GSR measurement in different places outside of a laboratory setting	Flexcomp physiological monitoring and a wrist-worn GSR sensor module developed by the authors	Palmar and distal forearm	Pearson’s correlation coefficients
Kasos et al. [32]	Assessing the similarities and differences in EDA measured at alternate and traditional anatomical sites	Obimon EDA	Fingers, feet, wrists, shoulders, and calves	Pearson’s correlation coefficient

**Table 2:** Sampling characteristics of the sensors and their place of measurement.

Sensor	Unit	Sample Rate	Measurement Place
Shimmer GSR 1	µSiemens (µS)	128 Hz	Left finger
Shimmer GSR 2	μSiemens (µS)	128 Hz	Right foot
Empatica 1	μSiemens (µS)	4 Hz	Left wrist
Empatica 2	μSiemens (µS)	4 Hz	Right wrist

**Table 3:** Descriptions of the pleasant and unpleasant stimuli video clips.

Time Slots	Emotion	Stimulus Description
0–32 s	Pleasant	Intro beach
33–34 s	-	Countback transition from pleasant to unpleasant
35–42 s	Unpleasant	Crash accident
43–58 s	Pleasant	Baby with lemon
59–60 s	-	Countback transition from pleasant to unpleasant
61–87 s	Unpleasant	Baby in building windows
88–109 s	Pleasant	Baby laughing
110–111 s	-	Countback transition from pleasant to unpleasant
112–172 s	Unpleasant	Baby jumping from a building
173–237 s	Pleasant	Puppies
238–239 s	-	Countback transition from pleasant to unpleasant
240–270 s	Unpleasant	Breaking bones
271–281 s	Pleasant	Looney Tunes end
282–285 s	Pleasant	Credits

**Table 4:** Statistics of the correlation matrices of each GSR signal.

Measurement Location	Correlation (*r*)	%	*p*-Value AS Test	*p*-Value K–S Test	Mean (*r*)	SD (*r*)	CV (*r*)
Right Wrist	Positive	61.8%	0.37	0.53	0.38	±0.25	0.65
	Negative	38.2%	0.53	0.52	–0.45	±0.27	0.61
Left Wrist	Positive	52.9%	0.004	0.02	0.59	±0.31	0.53
	Negative	47.1%	0.005	0.01	–0.61	±0.32	0.52
Right Foot	Positive	61.8%	0.59	0.64	0.42	±0.23	0.56
	Negative	38.2%	0.94	0.42	0.40	±0.23	0.57
Left Fingers	Positive	69.9%	0.87	0.90	0.45	±0.22	0.49
	Negative	30.1%	0.37	0.64	–0.34	±0.22	0.65

**Table 5:** GSR measurement range of each measurement location.

GSR Measure Range	Right Wrist	Left Wrist	Right Foot	Left Fingers
0–0.35 μS	88%			
0–0.75 μS		94%		
0.3–3 μS			29%	41%
3–10.7 μS			65%	53%

## Data Availability

The data presented in this study are available on request from the corresponding author. The data are not publicly available due to the written consent and confidentiality agreement regarding processing of personal data.
